# Investigation into the variation characteristics and influencing factors of coalbed methane gas content in deep coal seams

**DOI:** 10.1038/s41598-024-66011-2

**Published:** 2024-08-13

**Authors:** Qianya Zhu, Xuejia Du, Tong Zhang, Haiming Yu, Xiaobo Liu

**Affiliations:** 1 Department of Earth Sciences, Northeast Petroleum University, Daqing, 163318 China; 2 Department of Petroleum Engineering, University of Houston, Houston, TX 77204 USA; 3 Beijing AIDEJIAYE Technology & Development CO., Ltd, Beijing, 102206 China; 4 Department of Petroleum Engineering, Northeast Petroleum University, Daqing, 163318 China

**Keywords:** Coalbed methane, Deep coal seams, Reservoir properties, Gas content, Desorption and adsorption, Energy science and technology, Energy harvesting, Fossil fuels, Energy infrastructure

## Abstract

Gas saturation is a critical parameter for the selection and development of coalbed methane, as well as a key indicator reflecting the challenges in coalbed methane development and productivity evaluation of coalbed methane wells. As one of the significant factors influencing gas saturation, gas content plays a vital role in comprehensively investigating coal pore properties to fully comprehend the process and conditions of methane adsorption and desorption. In this study, 3^#^ and 15^#^ coals from Qinshui Basin, China was selected as research subjects. The experimental evaluation encompassed an examination of composition, pore characteristics, permeability characteristics of coal, rock mechanical parameters while discussing the impact of temperature and pressure on coal's adsorption and desorption capacity. The mineral characteristics analysis revealed that vitrinite is the main component with varying percentages and reflectance values in both 3^#^ and 15^#^ coal seams. The gas content and methane concentration in the 15^#^ coal seam are higher than those in the 3^#^ coal seam. The relationship between gas content within a coal seam and burial depth depends on achieving a balance between positive pressure effects caused by overburden stress exertion on gases trapped within pores under high pressures during burial history versus negative temperature effects due to cooling during geological processes over time. Predictions were made regarding deep-coal gas content which holds significant implications for accurately understanding variations in desorption behavior along with optimizing fracturing engineering.

## Introduction

Coalbed methane (CBM), also known as coal seam gas, is a significant mineral resource that coexists with coal. During its formation, when coal is shallowly buried, bacterial decomposition produces some methane gas. However, as burial depth and formation temperature and pressure increase, the organic matter in coal undergoes thermal degradation reactions and generates a substantial amount of methane gas. Therefore, this novel energy source called coalbed methane differs from conventional oil and gas resources. Its composition and enrichment state depend on various factors including original lithology, burial depth, formation process, and formation conditions. It primarily consists of CH_4_ while containing trace amounts of other multi-carbon chain compounds.

The advantages of coal bed methane (CBM) include its high calorific value (33.1–33.8 MJ/m^3^), high recovery rate, and environmental friendliness. Moreover, CBM reserves are estimated to be approximately 275 trillion m^3^ globally, with extensive distribution. According to Chinese data, the estimated amount of CBM resources in shallow coal seams within 2000 m is 36.8 trillion m^3^, surpassing that of the United States^1–5^. However, current extraction levels remain low, indicating significant potential for future development. Additionally, mining CBM enables waste utilization and enhances mine production safety by mitigating greenhouse gas emissions resulting from methane release during mining operations.

After extensive research, it has been demonstrated that coal bed methane is an unconventional form of natural gas that is produced and stored within coal seams. Based on its presence in the coal seam, coal bed methane can be categorized into adsorbed gas, free gas, and dissolved gas^6–10^. Over 90% of underground coal bed methane primarily exists as adsorbed gas on the inner surface of micro pores within the coal seam. Therefore, in order to enhance the extraction and recovery rate of coal bed methane and maximize its economic value, relevant technologies and methods must be employed to release it from the coal seam. The process of extracting surface mining for coal bed methane involves three stages: desorption where methane is released from pores; diffusion where methane spreads outside the pore; and permeation into fluid phase to achieve extraction of fluid containing methane^11–14^. Consequently, in order to eliminate adsorption under specific pressure conditions, pressure needs to be reduced; specifically lowered below the minimum pressure required by corresponding adsorbents for desorption to occur within the system and achieve desired results. Currently, mining processes mainly rely on drainage and depressurization methods by extracting bottom hole liquid to reduce internal system pressure until it falls below the minimum threshold necessary for successful separation of methane.

The production of gas from coalbed methane wells is closely correlated with factors such as the degree of coalbed methane enrichment, reservoir permeability, reservoir modifiability, and the water content of coal-bearing formations. In the assessment of coalbed methane resources in Sydney Basin coals by Faiz et al.^15^, they meticulously examined the physical characteristics of coal, encompassing density, porosity, oxygen content, etc., while delving into the burial history of coal to gain a comprehensive understanding of the formation process of coalbed methane reservoirs. Furthermore, they thoroughly considered the influence of geological background on these reservoirs, including factors such as geological structure and sedimentary environment. These aspects are pivotal for comprehending the attributes and potentiality of coalbed methane reservoirs. Simultaneously, an exhaustive analysis was conducted on secondary biogas reservoir characteristics which facilitated a more precise evaluation of natural gas content within coalbed methane reservoirs. Based on the consideration of the influence of geological structure and coal seam characteristics on coal seam gas content, Hemza et al. in addition to measuring coal seam gas content, also conducted the determination of basic chemical and physical parameters, including microscopic composition, specific element analysis, and in some cases other analysis. These parameters are closely related to the geological environment of the sampling area^16^. Zhang et al.^33^ analyzed the characteristics and influencing factors of coalbed methane well production based on the fundamental geological data and production data of coalbed methane wells in the Shouyang Block, with identification of the primary controlling factors accomplished through employment of the grey correlation method. They provided theoretical support and practical guidance for efficient coalbed methane development^17^.

Despite the challenges presented by low saturation, low permeability, strong anisotropy, and the soft coal characteristics of Chinese coal seams, significant progress has yet to be made in constructing a single hydraulic fracturing surface well for simultaneous pre-drainage of coalbed methane (CBM) from multiple seams under varying gas pressure systems. Hu et al.^18^ advocated the selection of coal seam gas vertical wells for hydraulic fracturing in geological structures and proposed a three-dimensional hydraulic fracturing technology characterized by pressure control, flow control, and sand rate control. The average gas yield and maximum gas yield of the test wells were 5.67 times and 12.88 times higher than those of conventional wells, respectively, achieving favorable drainage and production outcomes^18^. Wang et al.^19^ proposed a novel technology for enhancing deep coal seam gas reservoirs, which integrates efficient coal mining and gas extraction. Initially, the most suitable coal seam for hydraulic fracturing was determined based on effectiveness and economic feasibility principles. Subsequently, during the extraction of the primary coal seam, adjacent seams were modified using a spatially integrated gas extraction method (a fusion of underground and surface coal seam gas extraction techniques) to facilitate effective depressurization gas extraction^19^. Zhao et al. (2021^20^) posited that the integrated hydraulic fracturing curve can serve as an indicator of the gas production efficiency in coal-bed gas reservoirs. They have indicated that the gas yield from coal-bed gas wells corresponding to different types of fracturing curves follows this ranking: static > declining > rising > fluctuating^20^. Dai et al.^21^ conducted hydraulic fracturing experiments to investigate the characteristics of coal-bed gas storage and monitored the long-term variations in gas concentration and flow following hydraulic fracturing. They examined the impact of hydraulic fracturing on enhancing coal-bed permeability.

In order to elucidate the influence of geological conditions on the gas content and geochemistry of deep coalbed methane (CBM) reservoirs, Wei et al.^22^ conducted a comprehensive analysis of gas content and geochemical composition in selected coal samples using canister desorption method, gas chromatography (GC), and gas chromatography-mass spectrometry (GC–MS). By integrating these findings with the CBM geological characteristics specific to the study area, it was determined that this region exhibits elevated levels of gas content, in-situ stress, reservoir temperature, multiple coal seams, as well as intricate mining conditions. This study holds significant practical implications for advancing both theoretical understanding and technological advancements in deep CBM exploitation. The profound comprehension of coal pore characteristics plays a crucial role in the comprehensive understanding of methane gas adsorption and desorption processes and conditions, as coalbed methane is primarily adsorbed within its internal pores^23,24^. Additionally, after desorption, methane gas accumulates in fissures and is extracted to the surface through the fissure system. Therefore, acquiring an in-depth understanding of the morphology of the coal seam fissure system holds significant importance for comprehending permeability, fracturing mining, and other aspects related to coalbed methane geology^25,26^.

Based on the aforementioned analysis, numerous scholars have conducted extensive research on the porosity structure, adsorption, desorption, diffusion, and percolation of coal. This has led to a multitude of significant findings and insights. The diffusion process of gas in coal matrix pores is significantly influenced by the size distribution of pores. Given the substantial differences in internal pore structure and geological storage environment among coal rock bodies, it is imperative to evaluate the characteristics of coal reservoirs and explore the influence laws of coalbed methane adsorption and desorption processes on them. However, there is a relative scarcity in quantitative analysis literature regarding temperature- and pressure-dependent coal reservoir characteristics as well as the influence laws of coalbed methane adsorption and desorption in deep coal reservoirs. Therefore, it is essential to enhance relevant work on predicting coalbed gas.

The present study focuses on the 3^#^ and 15^#^ coals in Qinshui Basin, China, with a comprehensive analysis and discussion of coal gas content based on its mineral composition, pore-permeability characteristics, and mechanical properties. Conducted experimental assessments to investigate the impact of high-temperature and high-pressure conditions in deep coal seams on gas adsorption capacity, aiming to elucidate variations in coal and rock adsorption and desorption capabilities under diverse temperature and pressure scenarios. A predictive model for coal seam gas content was developed, which holds significant implications for accurately comprehending the desorption and adsorption kinetics of coal seams as well as optimizing hydraulic fracturing operations.

## Coal reservoir characteristics

### Coal formation characteristics

Both coalbed methane and conventional oil/gas reservoirs have potential for yielding methane-rich natural gas; however, they exhibit distinct differences in geological properties and extraction methods. Understanding these disparities is essential for optimizing the fracturing process specific to coalbed methane. Geologically speaking, coalbed methane reservoirs are typically situated within sedimentary basins' coal seams, whereas conventional oil/gas reservoirs are found in porous rock formations like sandstone or limestone. This variation in host rock composition directly affects each type's permeability and porosity levels, thereby impacting the efficiency of gas extraction techniques. Furthermore, extraction processes for coalbed methane differ significantly from those employed for conventional oil/gas. Coalbed methane requires specialized drilling techniques such as directional drilling or hydraulic fracturing to release trapped gases from its seams^27^. Conversely, traditional oil/gas reservoirs may be accessed through vertical wells with less reliance on hydraulic fracturing technology^28^.

#### Different storage mechanisms

In coalbed methane, the majority of methane gas is tightly adsorbed onto the inner surface of micropores due to high pressure and low temperature conditions within the coal seams. This adsorption process occurs as a result of interactions between the methane molecules and the carbonaceous material present in the coal matrix. Conversely, natural gas in conventional oil and gas reservoirs exists freely within non-coal pores, where it migrates through interconnected pore spaces and fractures. The disparity in gas storage mechanisms between these two types of reservoirs has significant implications for extraction methods and production efficiency. Understanding these distinct characteristics is crucial for optimizing recovery strategies and maximizing resource utilization in both coalbed methane and conventional oil and gas operations.

#### Different production mechanisms

Owing to distinct storage mechanisms, there are also notable variations in the production principles between these two sources. Coalbed methane recovery involves utilizing drainage depressurization as a method for extracting methane gas. This approach aims to reduce formation pore pressure by eliminating water from the formation. As soon as the formation pore pressure decreases to a specific value (equal to or lower than atmospheric pressure), desorption occurs, causing the previously adsorbed methane within coal seam pores to diffuse into lower-pressure water, partially dissolving therein before ultimately reaching the surface through water flow—Thus achieving natural gas recovery^29,30^. Conversely, conventional oil and gas recovery solely necessitates establishing an underground-to-surface channel via drilling, which creates a pressure differential enabling natural gas flow without desorption.

#### Different physical properties of reservoir rocks

There are differences in the reservoir characteristics of coalbed methane and sandstone, as shown in Table 1.

**Table 1:** Comparison of rock mechanics parameters of coal rock and sandstone.

Category		Modulus of elasticity GPa	Poisson’s ratio	Compressive strength, MPa	Tensile strength MPa
Sandstone	Scope	10–60	0.1–0.38	15–280	3.5–24
	General	–	–	500–100	5–12
	Average	35	0.24	75	8.5
Coal rock	Scope	0.3–2.7	0.1–0.6	5–60	1–6
	General	–	–	10–15	2–3
	Average	1.5	0.35	12.5	2.5

Coalbed methane is mainly enriched in coal, while conventional natural gas is mainly distributed in the pores of non-coal sedimentary rocks. The elastic modulus, compressive strength, and tensile strength of coal rock are all significantly lower than those of sandstone, and its Poisson's ratio is also noticeably higher than that of sandstone. This difference can be attributed to the contrasting mineral composition and structural characteristics between coal rock and sandstone. Coal rock primarily consists of carbonaceous minerals with a small amount of silicate minerals, resulting in a relatively loose structure and inferior mechanical properties. In contrast, sandstone is predominantly composed of dense silicate minerals such as quartz, feldspar, and mica, leading to superior mechanical properties.

#### Different properties of the fluid used for mining

In addition to the requirement of methane gas desorption, mining under low temperature and low-pressure conditions also necessitates careful consideration of the equipment used. Specialized machinery and tools are often required to withstand these extreme environmental factors while ensuring efficient extraction processes. Furthermore, the selection of suitable materials for construction and insulation becomes crucial in order to maintain operational integrity. Conversely, conventional oil and gas reservoirs do not impose such stringent demands on equipment due to their relatively stable temperature and pressure conditions. This allows for a wider range of standard industry practices and technologies to be employed without the need for specialized adaptations or modifications. As a result, operations in conventional reservoirs can often proceed with greater ease and efficiency compared to those in environments with more demanding extraction requirements.

#### Different displacement mechanism

In order to extract methane gas, it must first be desorbed, which requires reducing the pressure in the formation. When the pressure inside the coal seam is reduced by drainage methods, the adsorbed methane gas in the pores is desorbed and the gas is used to drive water flow. While in conventional oil and gas reservoirs, water is needed to drive gas to complete the mining.

### Coal rock characteristics

Coal is a complex porous material, with pores playing a crucial role in its structure. It is characterized by the presence of numerous pores. Unlike conventional oil and gas reservoirs, coal seams exhibit a dual pore structure comprising endogenous fractures and exogenous joints. Endogenous fractures, also known as cleavage, can be further classified into face cleavage and end cleavage^31^. In this study, the research focuses on the 3^#^ and 15^#^ coal reservoir rocks located in the Shizhuang Block of Qinshui Basin, China. Based on available parameter well samples and test reports, it has been determined that within the study area: the 3^#^ coal seam consists of humic anthracite exhibiting macroscopic characteristics such as black coloration, diamond-like luster, and silky gloss; while the 15^#^ coal seam is also black with diamond-like luster but semi-bright in appearance due to its predominant composition of albite along with secondary components including vitrinite, ophiolite, and fibrocarbon.

#### Microscopic composition and mineral determination

The micro-composition of 3^#^ coal seam is predominantly composed of vitrinite, with a content ranging from 56.9 to 85.7%, and an average value of 69.67%. The mineral content ranges from 1.8 to 17.2%, with an average value of 7.9%. Among these minerals, the clay mineral content varies between 1.1 and 16.3%, carbonate minerals range from 0.3 to 1.9%, and oxide minerals range from 0.1 to 0.7%. The micro-composition of 15^#^ coal seam is primarily comprised of vitrinite, with a content ranging from 61.2 to 85.7% and an average value of 74.2%. The mineral content ranges from 5.9 to 24.8% with an average value of 13.2%. Among these minerals, the clay mineral content varies between 4.9 and 21.3%, carbonate minerals range from 0.2 to 1.2%, and oxide minerals range from 0.2 to 0.8%.

#### Vitrinite reflectance of coal

3^#^ Coal seam: The distribution range is 2.78–3.69%, with an average value of 3.13%. Based on the reflectance values, it can be determined that the 3^#^ coal seam is in the high metamorphic stage of anthracite type III.

15^#^ Coal seam: The distribution range is 2.32–3.74%, with an average value of 3.17%. Based on the reflectance values, it can be determined that the 15^#^ coal seam is in the anthracite type III.

#### Characteristics of coal seam structure

3^#^ coal seam: The coal body structure primarily consists of primary and cataclastic structures, occasionally accompanied by granular structures. Based on exploration data, the bottom of 3^#^ coal seam is found to contain approximately 1.1 m of soft coal.

15^#^ coal seam: The coal body structure mainly comprises primary and cataclastic structures, with the presence of granular structure.

### Porosity and permeability of coal reservoir

#### Testing principles and methods

##### Porosity

**Figure 1:**
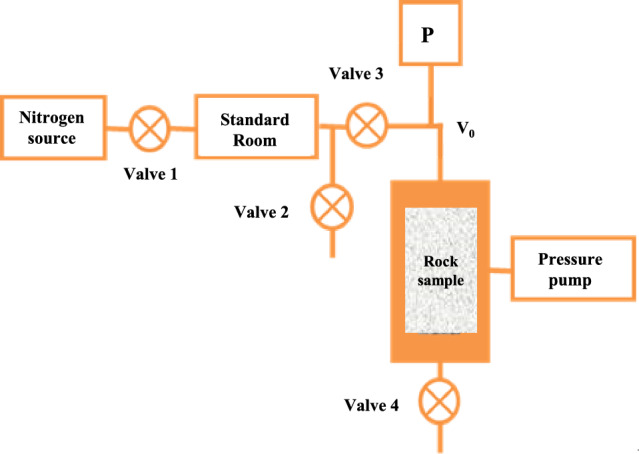
Schematic diagram of porosity test system for rock sample.

Porosity testing is conducted using Boyle’s law^32^, as shown in Fig. 1.

When the temperature is held constant, the volume of a specific mass of an ideal gas varies inversely with its absolute pressure.

1 $${P}_{1}{V}_{1}={P}_{2}{V}_{2}.$$

The formula was expanded to incorporate temperature fluctuations and non-ideal gas properties in order to ensure the precision of the test results.

2 $$\frac{{P}_{1}{V}_{1}}{{Z}_{1}{T}_{1}}=\frac{{P}_{2}{V}_{2}}{{Z}_{2}{T}_{2}}.$$

During the test, the test gas is initially introduced into the pore spaces of the rock sample, followed by allowing the system to achieve equilibrium. Subsequently, the gas diffuses into standard chamber *V*_1_, and porosity is determined through dual pressure calculations.

3 $${V}_{X1}={V}_{1}\frac{\frac{{P}_{2}}{{Z}_{2}{T}_{2}}}{\frac{{P}_{1}}{{Z}_{1}{T}_{1}}-\frac{{P}_{2}}{{Z}_{2}{T}_{2}}}-{V}_{0},$$

4 $$\varnothing =\frac{{V}_{X1}}{{V}_{d}},$$

where *P*_1_ system initial pressure, MPa. *P*_2_ system equilibrium pressure, MPa. *V*_0_ system apparent volume, cm^3^. *V*_1_ volume at initial pressure *P*_1_, cm^3^. *V*_2_ volume at equilibrium pressure *P*_2_, cm^3^. *Z*_1_ gas compression factor under pressure *P*_1_. *Z*_2_ gas compression factor under pressure *P*_2_. *V*_x1_ pore volume of rock samples, cm^3^. *V*_d_ rock sample apparent volume, cm^3^. *Φ* the porosity of the rock sample.

##### Permeability

The absolute permeability of rocks refers to the ability of a homogeneous fluid to flow through the pore space of the rock when it is completely saturated, without taking into account physical and chemical effects. Due to the relatively large pores in the reservoir, gas molecules cannot be fully adsorbed on the particle surface, so the rock's permeability determined by nitrogen measurement is very close to its absolute permeability.

According to the Darcy's formula^33^, the formula for calculating the gas permeability is shown as Eq. (5):

5 $$K=\frac{2{P}_{0}Q\mu L}{\text{A}({P}_{1}^{2}-{P}_{2}^{2})}\times {10}^{-1},$$

where A The cross-sectional area of the rock sample, cm^2^. L the length of the rock sample, cm. *μ* Gas viscosity mPa·s. ${P}_{1}$ Absolute pressure at the inlet gas, MPa. ${P}_{2}$ absolute pressure of the exhaust gas, MPa. *Q* gas flow, cm^3^/s. ${P}_{0}$ standard atmospheric pressure, 0.1 MPa.

#### Test results

##### Porosity

After measuring the true density and apparent density of coal, we can calculate that the porosity of 3^#^ coal seam is 4.03–6.18%, and that of 15^#^ coal seam is 4.97–6.38%.

##### Permeability

**Figure 2:**
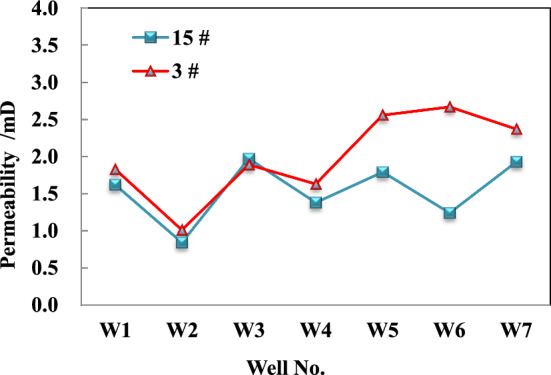
Interpretation results of well testing for different coal seam permeability in block wells.

The results of the injection/pressure drop test indicate that the coal seam in this area exhibits a high permeability. The measured permeability of 3^#^ coal seam ranges from 0.93 to 2.59 mD, while the permeability of 15^#^ coal seam is slightly lower than that of 3^#^ coal seam, ranging from 0.71 to 1.95 mD, attributed to its greater burial depth (Fig. 2).

##### Relative permeability of gas and water

**Figure 3:**
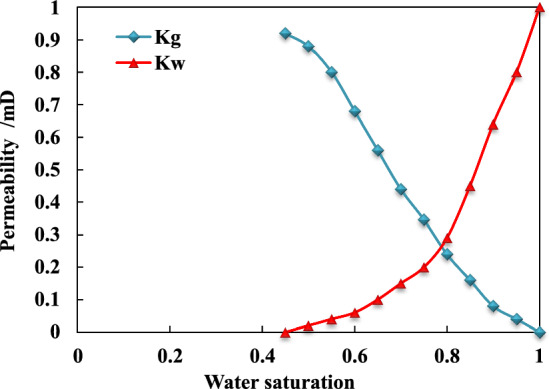
Permeability of gas and water phases in coal rock reservoirs.

The relative permeability can refer to the empirical data of Black Warrior Basin in the United States^34–36^, as shown in Fig. 3 below.

### Pressure and temperature characteristics of coal reservoir

#### Pressure

The analysis of injection/pressure drop test data from 6 wells in this area reveals that the pressure range of the 3^#^ coal reservoir is between 3.65 and 6.17 MPa, with a corresponding pressure coefficient ranging from 0.701 to 0.826, indicating an under pressured reservoir condition. Similarly, the pressure range for the 15^#^ coal reservoir falls within 4.27–6.83 MPa, with a pressure coefficient ranging from 0.694 to 0.831, also indicating an under pressured reservoir.

#### Temperature

The area is situated in the southern region of Qinshui Basin, characterized by an isothermal zone with a depth of approximately 55.0 m. It exhibits an average temperature of around 18 °C and showcases a relatively low geothermal gradient ranging between 1.7 to 2.3 °C/100 m.

## Gas bearing characteristics of coal

The coalbed methane content serves as a fundamental factor in the accumulation of coalbed methane, playing a crucial role in determining the production capacity and development potential of this valuable resource. It encompasses various aspects such as content, composition, gas saturation, resource enrichment degree, and spatiotemporal distribution characteristics. In the subsequent section, we will primarily delve into the gas content and saturation of coal seams along with their adsorption and desorption characteristics at significant depths.

### Amount of gas

The results of the coalbed methane gas content measurement indicate a high concentration of coalbed methane in this area. Specifically, the dry ash-free gas content of 3^#^ coal seam ranges from 8.7 to 21.6 m^3^/t, while the dry ash-free gas content of 15^#^ coal seam generally falls between 10.2 and 22.8 m^3^/t. Overall, the gas content of the 15^#^ coal seam surpasses that of the 3^#^ coal seam. Analysis of gas components reveals that methane is predominantly present in the coal seams within this area, accompanied by small amounts of carbon dioxide and nitrogen. More specifically, the methane concentration range for the 3^#^ coal seam spans from 78.67 to 96.12%, whereas for the 15^#^ coal seam it extends from 80.97 to 98.62%。

### Gas content saturation

Gas saturation is a crucial parameter for the evaluation and development of coalbed methane, as well as an important indicator reflecting the challenges in coalbed methane development and productivity assessment of coalbed methane wells. Gas content plays a significant role in determining gas saturation levels. Taking the southern part of Qinshui Basin as an example, there exists a strong negative correlation between gas saturation and burial depth of coal reservoirs, indicating that gas saturation decreases with increasing burial depth of coal seams. Generally, 3^#^ coal seam exhibits higher gas saturation compared to 15^#^ coal seam. Additionally, factors such as coal thickness, rank, and hydrogeological conditions also influence gas saturation levels. The study area demonstrates an increasing trend in burial depth from the basin's edge towards its center; however, there is no necessary relationship between gas content and gas saturation since the latter increases with greater burial depths. In the southern part of Qinshui Basin's main coal reservoirs, gas saturations range from 20.60 to 128.01%, with an average value of 70.53%. For 15^#^ coal seam specifically, typical values fall within the range of 21.39–98.70%, averaging at 59.47%. Gas saturations exhibit an east-to-west increase along with a south-to-north increment while being distinctly divided into three areas characterized by significant differences among them due to various influencing factors on this parameter including positive correlation with gas content but negative correlation with burial depth alongside certain associations with both coal thickness and rank levels present within this region situated in Qinshui Basin's annular slope belt where hydraulic plugging effect further enhances overall gas saturation.

## Adsorption and desorption characteristics of deep coal seam

### Adsorption of deep coal seam

#### Adsorption test

**Figure 4:**
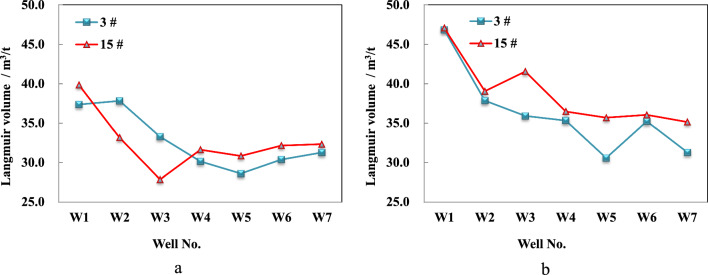
Langmuir volume of different coal seam in block wells under different experimental conditions. (**a**) Air dried basis, (**b**) Dry ash free.

**Figure 5:**
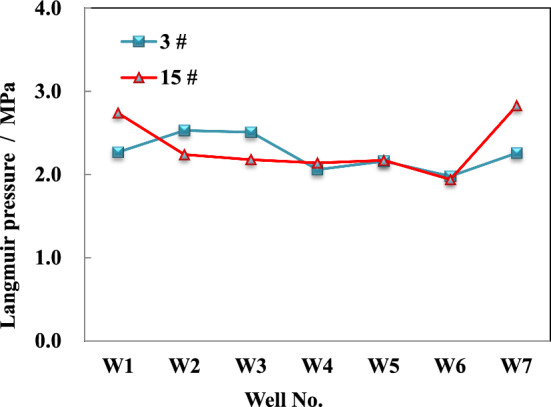
Langmuir pressure of different coal seam in block wells under different experimental conditions.

The isothermal adsorption experiments were conducted in 6 test wells for 3^#^ and 15^#^ coal seams, revealing that the maximum Langmuir volume adsorption capacity of the 3^#^ coal sample ranges from 30.14 to 46.82 m^3^/t, with an average value of 37.26 m^3^/t under clean ash-free conditions (Fig. 4). The corresponding Langmuir pressure range varies from 1.98 to 2.53 MPa, with an average value of 2.25 MPa (Fig. 5). Similarly, under the same conditions, the maximum Langmuir volume adsorption capacity of the 15^#^ coal sample ranges between 30.84 and 47.06 m^3^/t, with an average value of 39.57 m^3^/t. The corresponding pressure range is from 1.94 to 2.74 MPa, and its average value is 2.36 MPa. These experimental findings provide evidence for strong methane adsorption capacity in the main coal seams within this region.

#### Influence of temperature on maximum adsorption capacity of coal

The adsorption characteristics of coal seams are significantly influenced by deep high-temperature and high-pressure conditions, as coal is an organic rock highly sensitive to temperature and pressure. To investigate the relationship between adsorption in deep medium–high order coal reservoirs and temperature and pressure, four deep drilling samples were selected at J1 (2134 m), J2 (1967 m), J3 (1839 m), and J4 (1917 m). Isothermal adsorption tests were conducted under balanced water and high-pressure conditions. While most experiments on coalbed methane development focus below 1200 m with a constant temperature of 30 °C, this experiment employed 6 different constant temperature conditions: 30 °C, 35 °C, 40 °C, 45 °C, 50 °C and 55 °C to better understand the influence of varying temperatures on adsorption characteristics in a deep environment.

**Figure 6:**
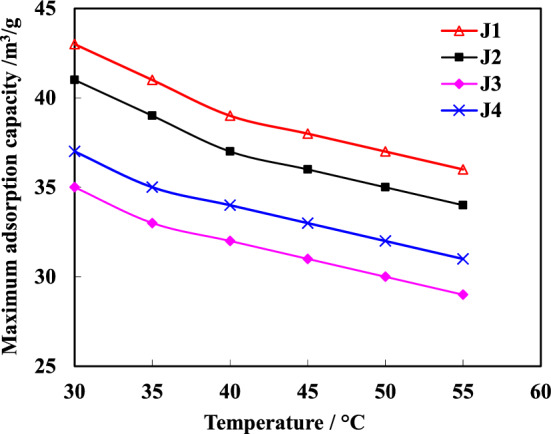
The variation pattern of maximum adsorption capacity of each rock sample with temperature.

The data presented in Fig. 6 indicates a gradual decrease in the maximum adsorption capacity of the same coal sample with increasing temperature. A linear decreasing trend is observed for the adsorption capacity of all samples at different temperatures. Notably, 3^#^ coal seam (J1, J3), which has a shallow burial depth, exhibits a strong linear fit and shows relatively rapid decay in its adsorption capacity with temperature. Based on the experimental results from four samples, it can be concluded that there is an average decrease of 0.25 cm^3^/g in the adsorption capacity of coal samples for every 1 °C increase.

#### Influence of pressure on maximum adsorption capacity of coal rock

**Figure 7:**
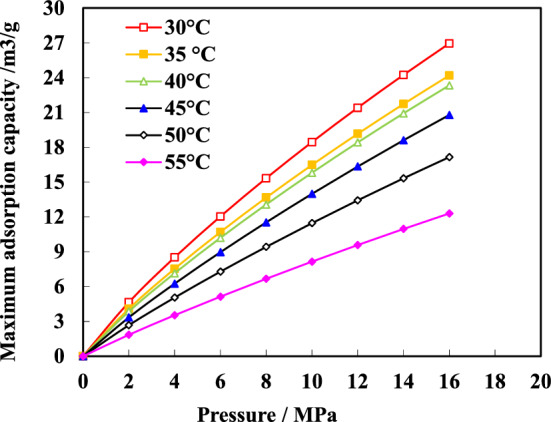
Isothermal adsorption curves of J2 rock sample at different temperatures.

The isothermal adsorption curve obtained from the experiment visually depicts the relationship between pressure and adsorption capacity. At five different temperatures, all samples exhibit consistent patterns in their isothermal adsorption curves. As pressure increases, there is a significant initial increase in adsorption capacity followed by gradual stabilization. Taking sample J1 (Fig. 7) as an example for analysis, coal shows rapid growth in methane adsorption capacity at low pressures; below 2 MPa, its adsorption capacity demonstrates nearly linear growth. With further increases in pressure, the rate of maximum adsorption capacity growth slows down and reaches approximately 90% when the pressure exceeds 8 MPa. Subsequently, under increasing pressure conditions, gas content within the coal seam significantly rises until it approaches its maximum value and stabilizes.

#### Influence of temperature on the Langmuir pressure

**Figure 8:**
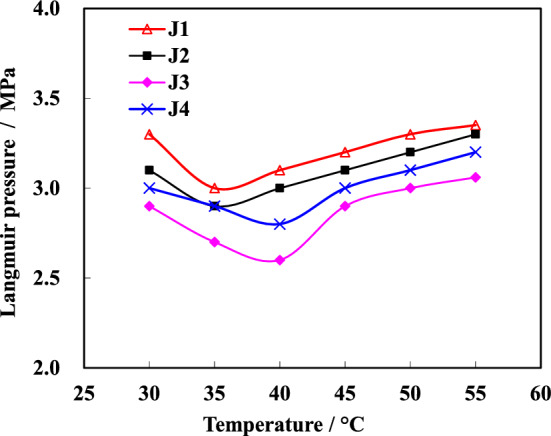
The variation pattern of Langmuir pressure of each rock sample with temperature.

The Langmuir pressure has a physical significance, referring to the pressure at which the adsorption capacity reaches half of its maximum value (i.e. Langmuir volume). A higher Langmuir pressure corresponds to a lower adsorption capacity. Figure 8 shows that, after synthesizing the experimental results of four samples, the Langmuir pressures of different samples exhibit a pattern of initially decreasing and then increasing with increasing temperature. The data reveal an inflection point between 35 and 40 °C during temperature rise. At this point, when the depth is shallow, the Langmuir pressure decreases with increasing depth; whereas when the depth is deep, it increases with increasing depth. This observed trend differs from previous studies' conclusions. Previously, it was believed that as temperature increased, Langmuir pressure exhibited a linear upward trend while coalbed methane's adsorption capacity gradually decreased. However, Fig. 8 demonstrates an inflection point in both temperature and corresponding depth within deep coal seams where optimal adsorption capacity exists. Based on these test results and considering geothermal conditions in the southern part of Qinshui Basin (Geothermal gradient: 2 °C/100 m. Surface isotherm thickness: 30 m. Surface temperature: 14 °C. Formation pressure gradient: nearly 1 MPa/100 m)^37,38^, it can be inferred that there exists an inflection point at approximately 1200 m depth corresponding to a temperature of 35 °C. In this area surrounding this position, the test values for Langmuir pressure are minimal while exhibiting relatively high adsorption capacities.

In summary, deep coal seams are exposed to high temperatures and pressures, with the increased pressure being a significant contributing factor to the enhanced adsorption capacity of coalbed methane with depth. On one hand, there exists a notable negative correlation between temperature and maximum adsorption capacity; on the other hand, at relatively large burial depths (> 1200 m), there is a positive correlation between temperature and Rankine pressure. This indicates that high-temperature environments can diminish the adsorption performance of coal seams, which hinders the enrichment of coalbed methane. However, during the development process, high-temperature environments have a beneficial impact on analyzing deep coalbed methane. Overall, experimental results demonstrate that the adsorption performance of deep medium-to-high-grade coal seams is influenced by both positive effects from pressure and negative effects from temperature. In this region, increasing pressure significantly enhances the adsorption performance of deep coal seams and holds considerable mining value.

### Desorption of deep coal seam

The desorption of coalbed methane (CBM) is commonly regarded as the inverse process of its adsorption. However, extensive drainage data and laboratory research results indicate that CBM desorption lags behind its adsorption process^39^. Adsorption and desorption are not completely synchronous, necessitating a correction to the gas desorption model. This study reveals that despite the lag in gas desorption, the desorption data points exhibit a behavior similar to the Langmuir adsorption model with changing pressure. Only when the pressure reaches zero does some residual gas remain unreleased. Therefore, an additional constant term "*c*" is introduced into the improved Langmuir equation to represent this residual gas quantity, resulting in the establishment of an enhanced desorption model as follows.

6 $$V=\frac{abP}{bP+1}+c,$$

where *a*, *b* and *c* are constants. *V* is the adsorption capacity of coalbed methane when the desorption pressure reaches *P*.

The above desorption model is fitted according to the desorption experimental data of coal samples, and the results are shown in Table 2.

**Table 2:** Fitting table of coal rock desorption model (CH_4_).

Sample	*R*_*o*_	Fitting formula	Correlation coefficient
No. 1	0.89	$V=\frac{5.79p}{0.45p+1}+1.83$	0.9938
No. 2	1.68	$V=\frac{13.64p}{0.88p+1}+3.65$	0.9913

**Figure 9:**
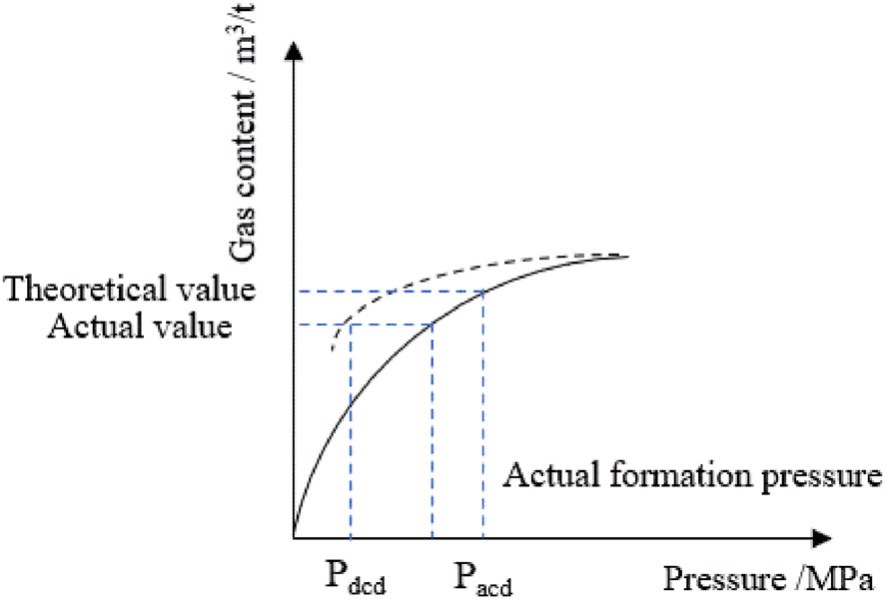
Critical desorption pressure and isothermal adsorption desorption curve of coalbed methane. *P*_acd_ Critical desorption pressure for adsorption curve calculation. *P*_dcd_ Critical desorption pressure for desorption curve calculation.

The correlation coefficients between the corrected desorption model and the experimental data exceed 0.99, indicating that the model is suitable for simulating the gas desorption process in coal reservoirs. As coalbed methane desorption and adsorption are not synchronous, the desorption of coalbed methane follows the desorption curve rather than the adsorption curve. The critical desorption pressure of a coalbed methane well, calculated using the isothermal desorption line and gas saturation, is lower than that calculated using the isothermal adsorption line and gas saturation. Accurate calculation of the critical desorption pressure significantly impacts the drainage dynamics of coalbed methane, particularly for high rank reservoirs. As depicted in Fig. 9, the critical desorption pressure of a single reservoir coalbed methane well is lower than that calculated using the isothermal adsorption line and gas saturation.

### Prediction of gas content in deep coal reservoir

The relationship between the gas content of deep coal seams and various factors is a complex interplay of variables that significantly impact the quality and quantity of the gas trapped within these coal deposits. Factors such as burial depth, coal grade, pressure gradient, and geothermal gradient individually and collectively exert unique influences on the gas content of deep coal seams. The gas content of deep coal seams is not static; rather, it exhibits two fundamental characteristics that delineate its behavior under different conditions. Firstly, it has been observed that under constant geothermal gradient conditions, an increase in coal grade results in elevated gas content at the same burial depth. This suggests that higher grades of coal have a direct impact on the amount of gas stored within them. Secondly, for a given coal grade, there is a "critical depth" where the relationship between gas content and burial depth changes—initially increasing to a certain extent before subsequently decreasing. This suggests that there exists a critical depth below which the gas content of the coal seam starts to diminish irrespective of its quality. This phenomenon may be ascribed to adverse pressure and temperature conditions at greater burial depths, which could impede gas retention within the seam. Moreover, any alteration in one factor affecting the gas content can have ripple effects on others. For example, under constant conditions elsewhere, an increase in geothermal gradient results in a reduction in critical depth, indicating that higher temperatures facilitate easier release of gases from the seam. Conversely, an increase in pressure gradient raises the critical depth, signifying that higher pressures assist retention within deeply buried coal reservoirs.

## Conclusions

The geological characteristics of coal seam gas attachments have been elucidated, and a comprehensive understanding of the mineral characteristics of 3^#^ and 15^#^ coal seams has been achieved. Bright coal is the primary component, followed by vitrinite, dark coal, and semi coherent coal. The microscopic composition of 3^#^ coal seam is predominantly vitrinite, with a content ranging from 56.9 to 85.7%, averaging approximately 69.67%. Additionally, the distribution range of vitrinite reflectance spans from 2.78 to 3.69%, with an average value around 3.13%. In the case of 15^#^ coal seam, the microscopic composition is also dominated by vitrinite, with a content ranging from 61.2 to 85.7%, averaging about 74.2%. The distribution range of vitrinite reflectance falls between 2.32 and 3.74%, averaging about 3.17%.

The adsorption of coal seam gas and the gas content characteristics of deep coal seams under temperature and pressure conditions have been elucidated. The dry ash-free gas content of the 3^#^ coal seam ranges from 8.7 to 21.6 m^3^/t, while that of the 15^#^ coal seam generally falls between 10.2 and 22.8 m^3^/t. Overall, the gas content of the 15^#^ coal seam exceeds that of the 3^#^ coal seam. Analysis of gas components reveals that methane is predominantly present in these coal seams, accompanied by small amounts of CO_2_ and N_2_. Specifically, for the 3^#^ coal seam, methane concentration ranges from 78.67 to 96.12%, whereas for the 15^#^ coal seam, it extends from 80.97 to 98.62%.

The established model for predicting deep coal seam gas content indicates that the relationship between positive pressure effect and negative temperature effect is the key factor controlling the correlation between coal seam gas content and depth. When the positive pressure effect dominates, there is a positive correlation between coal seam gas content and depth; when the negative temperature effect dominates, there is a negative correlation; when the positive pressure effect equals the negative temperature effect, coal seam gas content remains constant with depth.

## Data Availability

The datasets used and/or analyzed during the current study available from the corresponding author on reasonable request.
